# Sequential Cross-linking of Gallic Acid-Functionalized GelMA-Based Bioinks with Enhanced Printability for Extrusion-Based 3D Bioprinting

**DOI:** 10.1021/acs.biomac.2c01418

**Published:** 2022-12-22

**Authors:** Hatai Jongprasitkul, Sanna Turunen, Vijay Singh Parihar, Minna Kellomäki

**Affiliations:** † Biomaterials and Tissue Engineering Group, BioMediTech, Faculty of Medicine and Health Technology, Tampere University, Tampere33720, Finland; ‡ Brinter Ltd, Turku20520, Finland

## Abstract

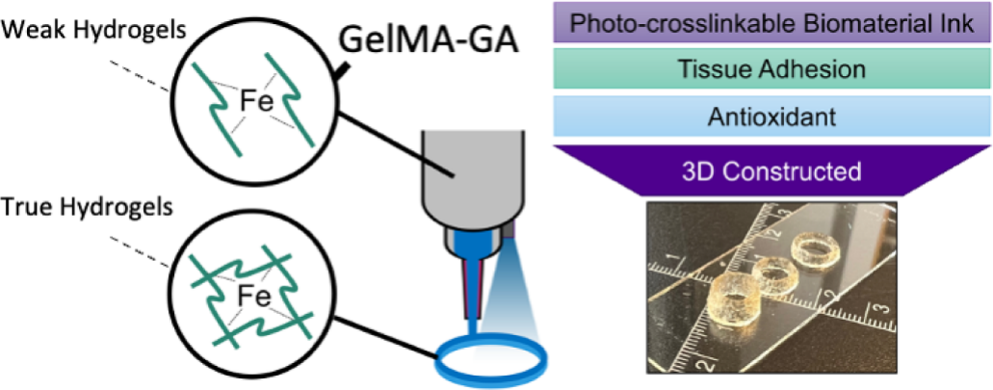

The printability of a photocross-linkable methacrylated gelatin (GelMA) bioink with an extrusion-based 3D bioprinter is highly affected by the polymer concentration and printing temperature. In this work, we developed a gallic acid (GA)-functionalized GelMA ink to improve the printability at room and physiological temperatures and to enable tissue adhesion and antioxidant properties. We introduced a sequential cross-linking approach using catechol–Fe^3+^ chelation, followed by photocross-linking. The results show that the ink formulation with 0.5% (w/v) Fe^3+^ in GelMA (30% modification) with 10% GA (GelMA30GA-5Fe) provided the optimum printability, shape fidelity, and structural integrity. The dual network inside the printed constructs significantly enhanced the viscoelastic properties. Printed cylinders were evaluated for their printing accuracy. The printed structures of GelMA30GA-5Fe provided high stability in physiological conditions over a month. In addition, the optimized ink also offered good tissue adhesion and antioxidant property. This catechol-based sequential cross-linking method could be adopted for the fabrication of other single-polymer bioinks.

## Introduction

3D bioprinting technologies are creating versatility in tissue engineering applications, such as using 3D tissue constructs as scaffolds, wound repairing, disease modeling, and organ-on-chip applications. Methacrylated gelatin (GelMA) has gained wide attention for mimicking the extracellular matrix,^1−4^ as GelMA can form hydrogels under UV light in the presence of a photoinitiator. Recently, GelMA has been one of the most used choices for bioinks to build 3D constructs using additive manufacturing. GelMA-based bioinks harness excellent biological properties and tunability that are preferential for 3D cell culture, including the skin, muscle, and cartilage.^5^ However, GelMA is difficult to form into complex 3D structures at room temperature (RT) or at low concentrations.^6^ The printability of GelMA is highly dependent on the polymer concentration and printing temperature.^7^ Therefore, more attention should be given to improving the printability and shape fidelity of GelMA bioinks because they allow the building of tissue-like constructs at high resolution.^6,8^ However, enhancing GelMA’s properties by increasing the concentration (>10%) leads to high cross-linking density and stiffness of the cured ink that adversely affects cell viability.^9^ Furthermore, printing at low temperatures for an extended time can also induce more cold injuries to cells and can cause irreversible cell damage.^10^ In addition, the cartridge, nozzle, and print-bed temperatures are not easily kept steady, which can lead to discontinuous extrusion. The most common way to improve GelMA’s printability is to incorporate other polymers, such as hyaluronic acid, alginate, gellan gum, chitosan, or synthetic polymers, to reinforce the hydrogel network.^9,11^ On the other hand, combining the bioink with other materials is not always ideal. It can cause unnecessary complexity and increase bioink’s preparation time, as reported in several publications studying single-component bioinks.^12^ In recent years, several stand-alone bioinks, formed with different chemical modifications and cross-linking techniques, have been explored to maximize the printability in extrusion-based 3D bioprinting.^13^ Dopamine-functionalized biopolymers and catechol-based biomaterials have been extensively explored as they mimic mussel adhesive protein that provides adhesion on wet tissue surfaces.^14−16^ It is noteworthy to mention that the hydrogel-based scaffolds with tissue adhesive properties help in the integration with surrounding tissue surfaces.

Gallic acid (GA) is a polyhydroxy aromatic compound with three phenol units, which are well known as catechol moieties. GA is also recognized for its tissue adhesive properties and antioxidant activity.^17^ However, according to our own findings, GA alone could not improve the rheological behavior of the GelMA ink as the printability of GelMA and GA-functionalized GelMA (GelMAGA) appears similar. Catechol-functionalized polymers with metal ions have been proven to have rapid network formation, pH-tunable cross-linking, and self-healing activity.^15,18^ The concentration of metal ions and pH can be used to precisely control the polymer network and their rheological properties.^16^ However, only a few studies have reported the application of GA–metal ion coordination chemistry to obtain printable biomaterial inks.^17,19−21^

**Figure 1:**
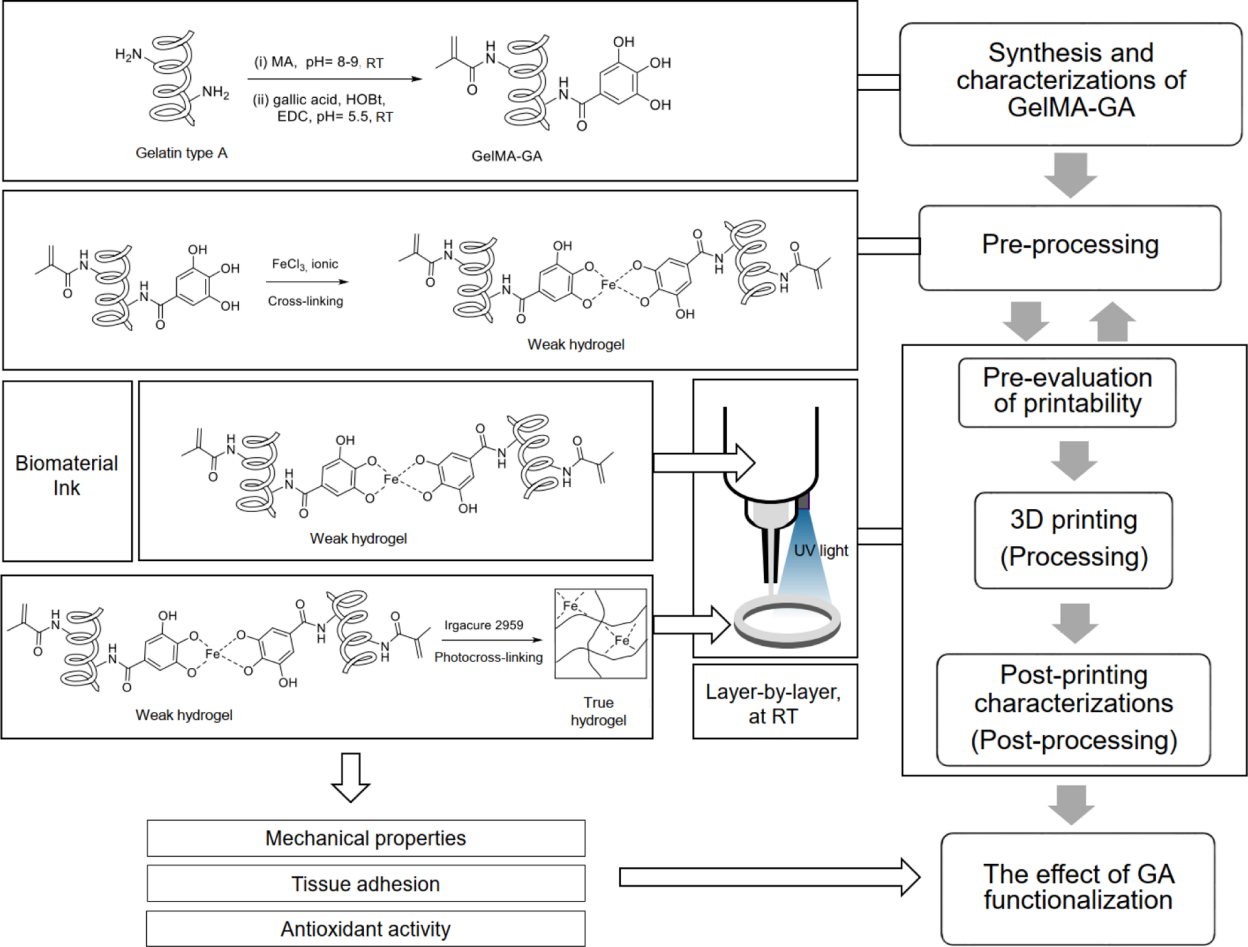
This schematic contains the set of biomaterial ink characterizations. (1) Synthesis and characterization of GelMAGA biomaterial inks, (2) preprocessing: optimization of different ink formulations, influenced by catechol–Fe^3+^ chelation, (3) pre-evaluation of printability in terms of Pr value and stackability, (4) processing: 3D printing and photocross-linking, (5) post-printing characterizations: 3D printing accuracy and structural integrity, and (6) the effect of GA functionalization: tissue adhesive, antioxidant, and mechanical properties. Weak hydrogels = viscous enough to be extrudable. True hydrogels = mechanically stable enough to maintain the structural integrity after printing.^24^

In this work, we functionalized gelatin with methacrylate (MA) and GA to create printable biomaterial inks by modulating the viscosity of the precursor using catechol/iron complexation. We hypothesized that the addition of a pre-cross-linker into a low-concentration GelMAGA could improve the printability and initial shape fidelity at RT/physiological temperature. Therefore, we propose a sequential cross-linking strategy by introducing two types of cross-linking techniques: catechol–Fe^3+^ chelation, followed by photocross-linking. The interactions between a gallate-tethered cationic polymer and metal ions resulted in shear-thinning behavior.^18,21^ The optimization was done by pre-cross-linking GelMAGA (5% w/v) with iron(III) chloride (FeCl_3_) with varying concentrations of FeCl_3_ (0.25, 0.5, and 1% w/v) to create a weak extrudable hydrogel. The pre-cross-linking strategies have been widely used in alginate and gellan gum-based bioinks.^22,23^ Moreover, GA (10% degree of modification)-functionalized GelMA can enhance tissue adhesion and offer antioxidant properties. Our study contains a set of biomaterial ink characterizations, as shown in Figure 1: (1) synthesis of biomaterial inks, (2) pre-processing, (3) pre-evaluation, (4) processing (3D printing), (5) post-printing characterizations, and (6) the effect of additional functionalization (tissue adhesive, antioxidant, and mechanical properties). In the pre-processing phase, the rheology of the ink formulations (GelMAGA and FeCl_3_) was measured, and a fiber formation test was performed to optimize the ink composition before printing. A printable set of inks were obtained with appropriate viscosity and rheological profiles. The pre-evaluation method was applied to further assess the printability of pre-cross-linked hydrogels with respect to the geometry of the printed constructs. In the processing step, layer-by-layer UV photocross-linking was used after printing to ensure the shape fidelity of the 3D constructs. In post-printing characterizations, the accuracy of printed constructs was measured. Long-term stability of the printed structures was observed in the incubated environment (swelling and dissolution test). The effect of grafting the GA onto GelMA was evaluated by oscillatory measurement (mechanical properties), tack test (tissue adhesion), and antioxidant activity.

## Materials and Methods

### Materials

Gelatin type A (300 bloom strength, porcine skin), methacrylic anhydride, GA (3,4,5-trihydroxybenzoic acid), 1-ethyl-3-(3-dimethylaminopropyl)-carbodiimide hydrochloride (EDC), 1-hydroxybenzotriazole hydrate (HOBt), glycine, trinitrobenzene sulfonic acid (TNBS), FeCl_3_, and Irgacure 2959 (I2959) were purchased from Merck KGaA, Darmstadt, Germany. A dialysis membrane with a molecular weight cutoff (MWCO) of 14 kDa was purchased from Spectra/Por, Repligen Corp., USA. DI water (deionized water, Miele Aqua Purificator G 7795, Siemens) and u.p. water (ultra-pure, Sartorius Arium Mini, 0.055 μS/cm) were used. Dulbecco’s phosphate-buffered saline (DPBS) was prepared in the laboratory. All solvents were of analytical quality.

### Synthesis of Biomaterial Inks (GelMA and GelMAGA)

GelMAGA was synthesized using a two-step reaction. GelMA batches with 30 and 60% degrees of methacrylation were synthesized as previously described^3,7^*via* nucleophilic reaction of residual amine on gelatin molecules and methacrylic anhydride. Briefly, 10% w/v of gelatin type A was dissolved in DPBS under the basic conditions (pH 9) at 60 °C. Next, methacrylic anhydride was added dropwise, and the degree of functionalization was controlled by varying the ratio of gelatin and methacrylic anhydride in each modification. The pH was maintained at 9 after each addition of methacrylic anhydride. The reaction was carried out at 50 °C for 3 h. After completion, the reaction mixture was dialyzed with a 14 kDa MWCO membrane at 40 °C against DI water for 72 h (water was changed twice daily). GelMA was then lyophilized and stored at 4 °C. The degree of methacrylation was confirmed using UV-spectral measurement (Shimadzu UV-3600 plus UV–vis–NIR spectrophotometer).^3,25^ After that, GelMA30 and GelMA60 were functionalized with GA using the protocol reported previously.^26^ GelMAGA was synthesized *via* a carbodiimide coupling reaction using EDC. HOBt was added (1 equiv) with respect to GelMA. The degree of GA functionalization was controlled using EDC (0.2 equiv). The reaction was let to proceed for 4 h (pH 5). The unreacted GA and EDC were removed by dialysis (MWCO = 3500 Da, regenerated cellulose membrane) in 1 M NaCl (pH 5.3) at 4 °C for 3 days, followed by dialysis against DI water for 24 h. At last, the solution was freeze-dried. The degree of GA was characterized by UV spectra. The calculation of the number of free amine groups in gelatin type A, GelMA, and GelMAGA was done using the calibration curve of the glycine standard^27^ (Figure S1).

### Preparation of Biomaterial Ink Formulations

GelMA and GelMAGA inks were dissolved at 5% w/v in a photoinitiator solution at 40 °C (Irgacure 2959, 0.5% w/v in DPBS) and stirred for 2 h until they were completely homogeneous. The pH was tuned to 7.5 to gain proper viscosity. The studied biomaterial ink formulations were GelMA, GelMAGA, and GelMAGA with a pre-cross-linker (FeCl_3_). Gelatin methacrylate with 30 and 60% degrees of modifications was named GelMA30 and GelMA60, respectively. GelMA30 and GelMA60 functionalized with GA were termed GelMA30GA and GelMA60GA, respectively. GelMA30GA and GelMA60GA with FeCl_3_ were GelMA30GA-xFe and GelMA60GA-xFe, respectively, where x indicated the concentration of FeCl_3_ (2.5 is 0.25% and 5 is 0.5% w/v). All the formulations are listed in Table 1.

**Table 1:** Biomaterial Ink Compositions with Various Modification Degrees of Methacrylate and GA and Fiber Quality^a^

formula	degree of methacrylation [%]	degree of GA modification [%]	FeCl_3_[% w/v]	fiber quality
GelMA30 (RT)	30	0	0	droplet
GelMA60 (RT)	60	0	0	droplet
GelMA30GA (RT)	30	10	0	droplet
GelMA60GA (RT)	60	10	0	droplet
GelMA30GA-2.5Fe (RT)	30	10	0.25	discontinuous fiber
GelMA60GA-2.5Fe (RT)	60	10	0.25	discontinuous fiber
GelMA30GA-5Fe (RT)	30	10	0.5	continuous fiber
GelMA30GA-5Fe (37 °C)	30	10	0.5	continuous fiber
GelMA60GA-5Fe (RT)	60	10	0.5	discontinuous fiber

a GelMA = gelatin methacrylate; GelMAGA = gelatin methacrylate functionalized with GA; GelMAGA-Fe = Fe^3+^ pre-cross-linked gelatin methacrylate functionalized with GA.

### Prescreening and Flow Behavior of Biomaterial Inks

Biomaterial inks were evaluated using the pre-processing method: formulation of inks, fiber formation, and rheological measurements to prescreen the printability without loading the ink into the 3D bioprinter. We followed the simple prescreening protocols published previously:^22^ filament formation and stackability test. The biomaterial inks with different formulations (Table 1) were loaded into a 10 mL cartridge and capped with a tapered UV-shielded nozzle (200 μm in diameter, Nordson EFD, Germany). The ink filament was formed in the air at RT (24 °C) and at 37 °C by an automatic dispenser, and a video was recorded simultaneously with a camera. The ink compositions were chosen based on filament characteristics. The filament was deposited on the glass surface to investigate the stackability.

The flow behavior of different ink formulations was evaluated by a rotational rheometer (Discovery HR-2, TA Instruments Inc., USA) with a plate–plate geometry (12 mm in diameter, a gap size of 2.5 mm). The measurements were recorded at RT. The rheological tests performed were temperature ramp (viscosity–temperature), *in situ* photo-polymerization (gelation time), shear-thinning (viscosity–shear rate), yield stress, and recovery behavior. The temperature-dependent behavior was measured with a temperature sweep varying from 40 to 4 °C at the rate of 2 °C/min. The gelation times of the inks were determined *via in situ* photo-polymerization using a rheometer with an external UV source (BlueWave 50 UV curing spot lamp, DYMAX Corp., USA). The UV intensity was measured using a power meter console (PM100USB, Thorlabs Inc., USA) coupled with an S310C thermal sensor. The estimation of UV light intensity as a function of the distance from the light source is described in Figure S2. Viscoelasticity was measured using oscillatory mode at RT as a function of time (500 s, a UV lamp at a wavelength of 365 nm and 25 mW/cm^2^ in UV intensity, UV light was activated at 100 s), while strain and frequency were kept constant at 1% and 1 Hz, respectively. The shear-thinning properties of the inks were also determined in flow mode, with the shear rate varying from 0.01 to 800 s^–1^. The shear-thinning coefficients and yield stress values were determined from the linear region of the graph using eqs 1 and 2, respectively. Shear-thinning coefficients were calculated using the power law, eq 1.
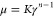
1

The flow behavior index *n* describes the shear-thinning ability of the ink. If *n* = 1, the ink follows Newtonian behavior. If *n* > 0.6, the material is weakly shear-thinning and if *n* ≤ 0.2, the ink has good shear-thinning properties and excellent printability.

Yield stress values were determined from the yield stress–shear rate plot, where the shear stress begins to increase from the intersection at the *Y*-axis, using the Herschel–Bulkley model (eq 2).
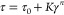
2where τ is the shear stress measured on the inks and τ_0_ is the yield stress. The yield point determines the flow initiation of the inks at the level of the applied shear stress.

The recovery behavior test (thixotropy) is to characterize the bioink’s ability to recover its viscosity after a high shear rate has been applied. The measurements were performed at a low shear rate (0.01 s^–1^ for 200 s) to simulate at-rest conditions before extrusion, followed by a high shear rate (500 s^–1^ for 100 s) to mimic shear forces in the nozzle tip during extrusion, and finally a low shear rate (0.01 s^–1^ for 200 s) to simulate bioink recovery after extrusion.

### Pre-Evaluation of Printability

After obtaining the best printable biomaterial ink formulation and optimal printing parameters, we assessed the structural integrity. The shape and stackability of the printed filament are the first parameters that can ensure a successful printing process and yield high printing resolution. Thus, the true printability was quantified by semi-quantitative measurement from the shape of the printed structures. Prescreened biomaterial ink formulations were loaded into a 10 mL cartridge (Optimum syringe barrels, Nordson EFD, USA) and transferred in an incubator (37 °C) for 30 min to remove any air bubbles. Next, the cartridge was installed into a multi-material 3D bioprinting platform (Brinter One, Brinter Ltd., Finland) by capping it with a 200 μm plastic UV-shielded tapered nozzle (SmoothFlow, Nordson EFD, USA). A pneumatically operated Pneuma Tool printhead was used for printing. The printing pressure was set according to the prescreening test results. Printing speed and printhead temperature were kept constant at 8 mm/s and RT, respectively. 3D constructs were printed using the layer-by-layer deposition approach, followed by photocross-linking to stabilize the structure (an integrated UV/vis LED module was used at a wavelength of 365 nm with 25 mW/cm^2^ intensity for 10 s for each layer and 60 s for the post-curing process).

As previously described,^22^ the printability assessment of different biomaterial ink compositions was done by printing two-layered grid patterns. The shape of the printed pores is evaluated using eq 3.
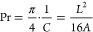
3in which *C* is the circularity of the enclosed pore, *L* is the perimeter, and *A* is the pore area. The printability (Pr) of the biomaterial ink compositions was determined by the squareness of the pores inside the grid structure. The Pr value of 1 indicates a perfect square shape. A computer-aided design (CAD) model for the square grids (20 × 20 × 0.4 mm^3^) was drawn with Autodesk Fusion 360 software and used as a standard for this assessment.

### 3D Printing

3D printed cylinders were used to evaluate the ability of the inks to support the weight of each layer while maintaining the printing resolution. We chose poloxamer (40% w/v, Kolliphor P 407, BASF Corp., USA) as a control printing material. It gave high geometric accuracy with minimal deviation compared to the CAD model. The prescreened ink was printed into cylinders (10 mm in outer diameter) with different heights (1.5, 2.5, and 5 mm). Each structure was cured in a layer-by-layer fashion using the bioprinter’s integrated UV/vis LED module at a wavelength of 365 nm with 25 mW/cm^2^ intensity for 10 s for each layer and 60 s for the post-curing process. The dimensions of the cylinders were measured from photos with ImageJ and compared with the printed control structure to determine the printing accuracy. The filaments of the prescreened inks and the control material were observed with a contact angle camera (Theta Lite, CMOS 1/2″ USB 3.0 digital camera with fixed zoom, a resolution of 1280 × 1024 pixels, Biolin Scientific, Sweden) to measure the width of the filaments (OneAttension v2.1).

### Tissue Adhesion Test

To observe the impact of GA on the adhesive properties of the ink, a tack test was performed for GelMA and GelMAGA using a rotational rheometer at RT. Chicken skins and porcine muscles (freshly purchased from the market) were harvested and glued to the 12 mm geometry, and the inks were placed on the bottom of the plate.^14,28^ After that, the geometry with animal tissue attached was moved in contact with the inks with a constant compressive force (0.1 N) for 120 s to establish a uniform molecular contact between the tissue and the ink. Subsequently, the inks were *in situ* photopolymerized with a UV lamp for 120 s. Thereafter, the geometry was pulled up at a constant velocity of 20 μm/s to record the change in axial force as a function of time. A graph was then plotted to observe the influence of GA in GelMA on adhesive properties. The harvested tissue was kept moist during the measurement.

### Antioxidant Activity

A 2,2-diphenylpicrylhydrazyl (DPPH) radical scavenging assay was used as a preliminary assessment of the changes in the antioxidant properties upon modification of GelMA with GA. The free radical scavenging activity of GelMAGA was evaluated using the DPPH method.^17,29^ GelMAGA was dissolved in DI water at 2 mg/1 mL concentration, followed by 1 mL of DPPH solution (1 mg/12 mL in methanol). After incubation at 25 °C for 30 min, the absorbance of the resulting solution was measured at 517 nm using a UV–vis spectrophotometer. The DPPH scavenging activity (%) is calculated from eq 4.
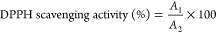
4where *A*_1_ is the absorbance of blank DPPH solution that was used under the same reaction conditions in the absence of synthesized polymers and *A*_2_ is the absorbance of DPPH solution in the presence of polymer samples.

### Viscoelastic Properties

To determine the effect of GA functionalization on mechanical properties, the oscillatory measurements were carried out in the linear viscoelastic region using an amplitude sweep (0.1–100% strain range and at a constant frequency of 1 Hz) and a frequency sweep (a frequency range of 0.1–100 Hz and at a constant strain of 1%). The biomaterial inks were cast in the molds (2.5 mm height, diameter of 12 mm) and were exposed to 365 nm UV light (25 mW/cm^2^) for 120 s. Each sample was placed between the 12 mm geometry and the platform with a gap size of 2.5 mm. Storage modulus (*G*′) and loss modulus (*G*″) were obtained from the slopes. After that, tan δ was calculated from *G*′ and *G*″ to determine the viscoelastic properties and plotted as a tan δ−strain curve.

For further in-depth structural analysis, the average mesh size and cross-linking density were determined from oscillatory measurement results.^30^ The average mesh size (ξ, nm) calculation was applied using the storage moduli (*G*′) of resulting hydrogels (the best formulation ink) at 120 s UV exposure time. Equation 5 estimates the average mesh size (ξ) of hydrogels at different exposure times
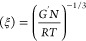
5where *G*′ is the storage modulus of the hydrogel, *N* is the Avogadro constant (6.023 × 10^23^ mol^–1^), *R* is the molar gas constant (8.314 J K^–1^ mol^–1^), and *T* is the temperature (298 K).

Moreover, cross-linking density (*n*_e_, mol/m^3^) of the hydrogels was calculated using the storage modulus from the linear region of the frequency sweep test. The data provided the total number of elastically active junction points in the network per unit of volume using eq 6.^30^
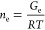
6where *G*_e_ is the average value of storage modulus from the linear region of oscillatory frequency sweep measurement.

### Stability Study

The chosen biomaterial ink was printed into 3D grid structures (10 × 10 × 5 mm^3^). Subsequently, an extra photocross-linking method was applied to the printed structures to gain an additional stability during the incubation.^11^ Briefly, the printed structures were immersed in DPBS containing 0.05% of Irgacure 2959 and exposed to UV light (10 mW/cm^2^) for 5 min. Post-stabilization, the printed samples were immersed in the solution (DI water, DPBS, or DMEM) at 37 °C. The structures were weighed at time points 0, 1, 2, 3, 5, 7, 15, and 30 d. At the zero time point, the samples were defined with a weight of *W*_0_. At every time point, the samples were removed from the solution and the excess solution from the surface was removed to obtain the *W*_s_. The swelling ratio was calculated as *W*_s_/*W*_0_.

### Statistical Analysis

The results of oscillatory measurements were presented as mean ± standard deviation (SD). The analysis was performed using Student’s *t*-test to determine the differences between groups, and the significance was defined at *p* < 0.05.

## Results

### Characterizations of Synthesized Biomaterial Inks

The biomaterial inks were synthesized with various modification degrees, as listed in Table 1. The degree of methacrylation of GelMA30 and GelMA60 was obtained as ∼31 ± 5% (∼0.09 mmol/g) and ∼64 ± 5% (∼0.18 mmol/g) (batch-to-batch variations), confirmed by the TNBSA assay (Figure S3). The degree of GA modification on GelMA was quantified using UV/vis absorption measurements (GA ∼10% or ∼0.03 mmol/g) (Figure S3). The degree of methacrylation and GA modification were calculated based on the measurements of free amines in modified gelatin with respect to unmodified gelatin, as shown in Table S1. The pH dependency of GA further confirmed the conjugation.^26^ GelMAGA solution turned brown at the basic condition (∼pH 8), indicating that GA functionalization was successful in the GelMA backbone (Figure S4).

### Prescreening of Bioink Formulations

The concentrations of biomaterial inks were set to 5% w/v in DPBS (0.5% w/v I2959). To obtain the high printability and stability at RT, pre-cross-linker FeCl_3_ was applied to GelMAGA using various concentrations. The biomaterial inks and the fiber quality were assessed as a function of methacrylation in GelMAGA and FeCl_3_ concentration, as shown in Table 1. The fiber quality was assessed from the fiber formation ability of the inks after being extruded from the nozzle. From Table 1, GelMA30, GelMA60, GelMA30GA, and GelMA60GA (5% w/v) at RT were extruded as droplets. GelMA30GA-2.5Fe and GelMA60-2.5GA could not form stable enough fiber during the extrusion at RT, as they hardly formed a continuous fiber. At RT and at 37 °C, GelMA30GA-5Fe produced approximately 5 cm long coherent filaments. However, GelMA60-5Fe produced irregular and discontinuous fiber. We also tuned the concentration of Fe^3+^ into 1% w/v in both GelMA30GA and GelMA60GA, but the inks were too gelated and clogged the nozzle.

### Flow Behavior of Biomaterial Inks

**Figure 2:**
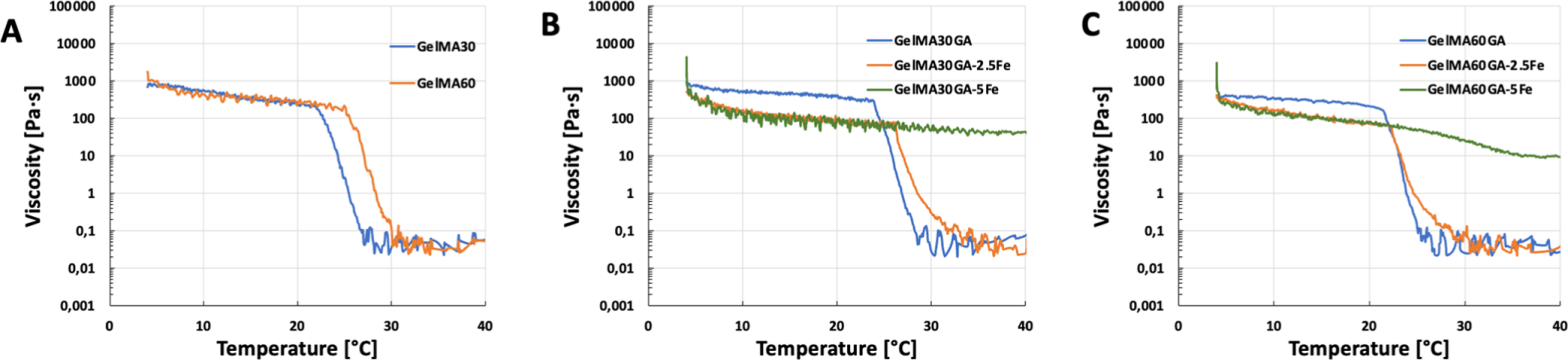
Rheological measurement of viscosity as a function of temperature. All samples were measured over the temperature range from 4 to 40 °C. (A) GelMA with 30 and 60% degrees of methacrylation, (B) GelMA30GA group with/without Fe^3+^, and (C) GelMA60GA group with/without Fe^3+^.

To further deepen the study of the ink properties, the flow behavior of the inks was measured in terms of viscosity as a function of temperature. Figure 2A–C presents the temperature dependence of viscosity between 4 and 40 °C. The viscosity of GelMA (Figure 2A) and GelMAGA (Figure 2B,C, blue and orange curves) without (or with 2.5Fe) additional cross-linker (FeCl_3_) decreased significantly after 25 °C, whereas GelMA30GA-5Fe and GelMA60GA-5Fe (Figure 2B,C, green curve) had steady viscosity levels, which only slowly fell after reaching 30 °C.

**Figure 3:**
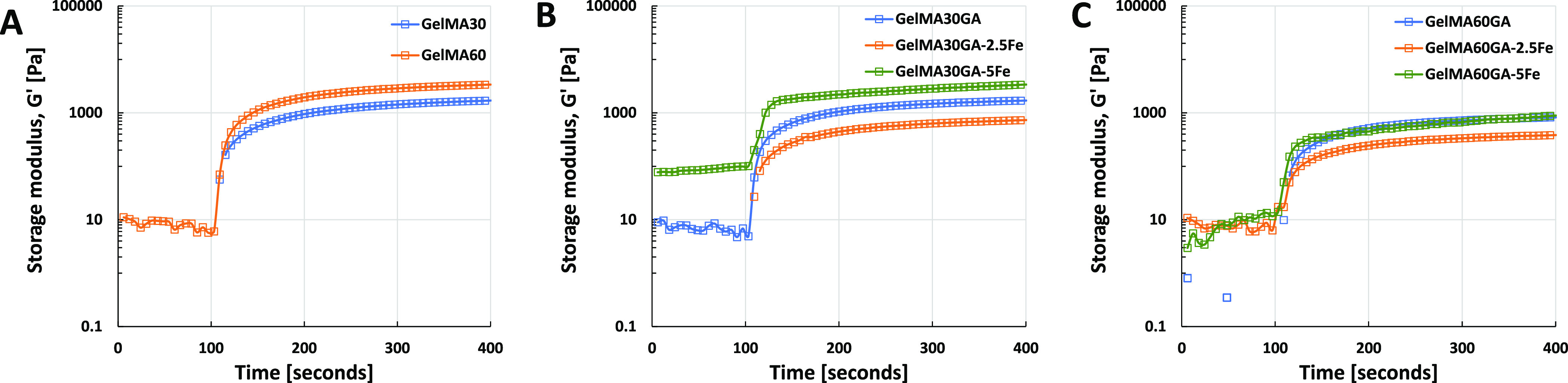
*In situ* photo-polymerization test to observe the gelation time of each ink formulation (time sweep of oscillatory measurement, 25 mW/cm^2^ for 300 s, at RT). (A) Pure GelMA30 and GelMA60, (B) GelMA30GA group with/without Fe^3+^, and (C) GelMA60GA group with/without Fe^3+^.

*In situ* photo-polymerization (Figure 3A–C) shows the gelation time of all ink formulations (storage modulus as a function of time). All inks showed an increase in storage modulus right after being exposed to UV light and reached the maximum cross-linking degree after 60 s. The gelation time and storage moduli of GA-functionalized GelMA did not differ from the pure GelMA. However, FeCl_3_ in GelMAGA required more than 60 s before the storage modulus reached the plateau.

**Figure 4:**
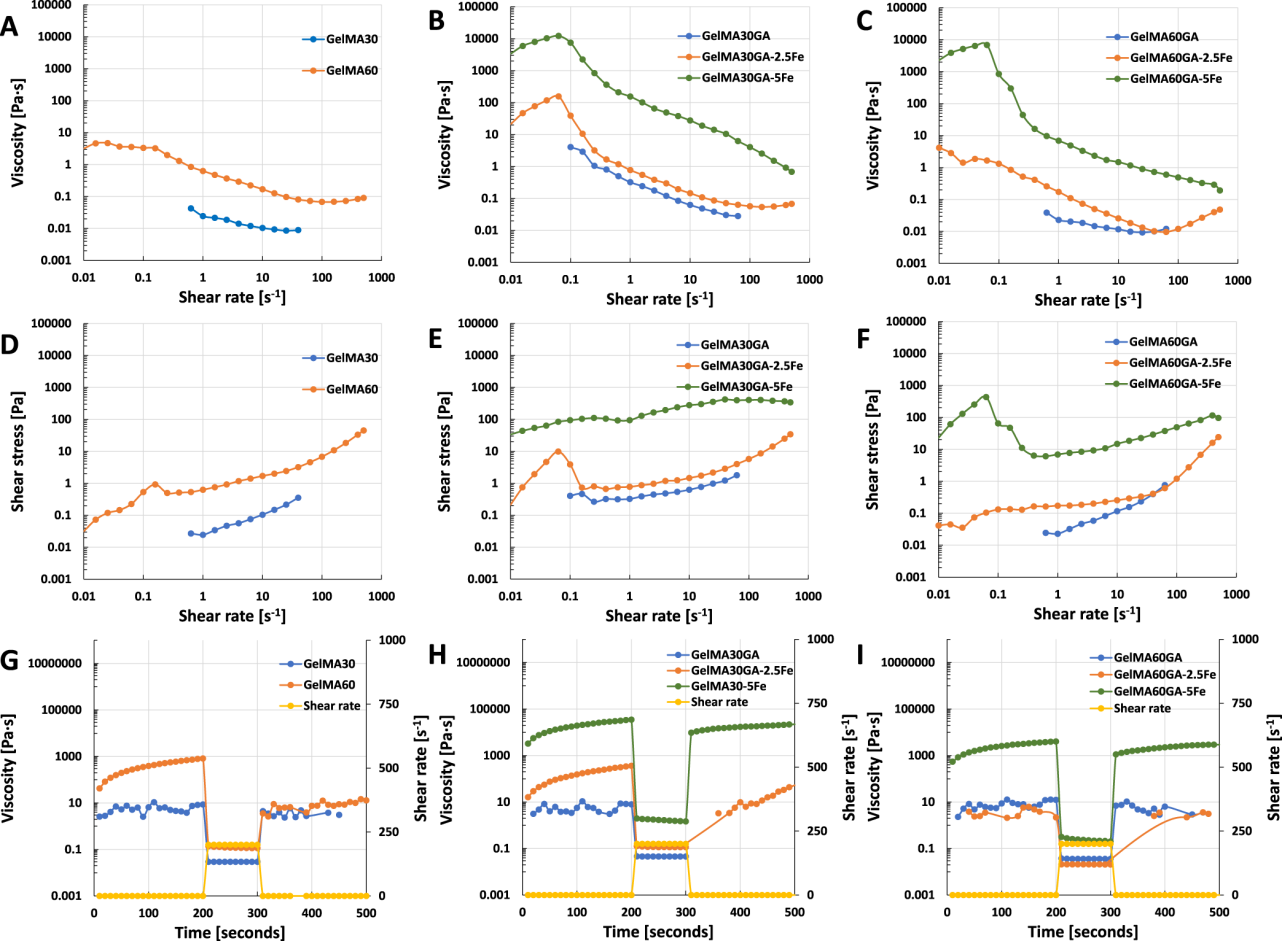
Rheological measurements in the flow mode: shear-thinning (A–C), yield stress (D–F), and recovery behavior (G–I) at RT. (A,D,G) Pure GelMA30 and GelMA60, (B,E,H) GelMA30GA group with/without Fe^3+^, and (C,F,I) GelMA60GA group with/without Fe^3+^.

Figure 4A–I presents the flow curve, shear-thinning, and recovery behavior of different ink formulations at 37 °C. All the ink formulations provided *n* < 1, which proves shear-thinning behavior. In detail, it was observed that GelMA30 and GelMA60 at RT have a weak shear-thinning ability because of low viscosity and low yield stress (Figure 4A,D,G). Shear-thinning coefficients of *n* > 0.6 also confirmed the results, and the prescreened results also showed droplet formation as the material was extruded out from the nozzle.

However, GA functionalization alone could not improve the shear-thinning behavior of the inks and showed almost similar results to GelMA (Figure 4, blue curves). The addition of 0.25 or 0.5% w/v of FeCl_3_ in GelMA30GA and GelMA60GA significantly improved viscosity, shear-thinning, yield stress, and recovery behavior. GelMA30GA-5Fe and GelMA60GA-5Fe had more obvious shear-thinning ability than GelMA30GA-2.5Fe and GelMA60GA-2.5Fe, as shown in Table 2. In addition, in Figure 4H,I and Table 2, GelMA30GA-5Fe and GelMA60GA-5Fe rapidly recovered back their viscosity (∼73 and 72% recovery) after removing the high shear rate. In comparison, 0.25% w/v FeCl_3_ inks could not recover their viscosity and permanently lost their properties (Figure 4B,C,E,F,H,I, orange curves). According to the curves (Figure 4C,F, green curves), the viscosity of GelMA60GA-5Fe had a sharp drop with an increasing shear rate (0.1 s^–1^), which correlates with the irregular shape of the extruded filaments.

**Table 2:** Flow Behavior of Each Ink Formulation: Viscosity, Shear-Thinning Coefficients, Yield Stress, and Recovery Rate during the Extrusion

compositions	*n*	viscosity [Pa·s]	*K*	**τ**_0_ [Pa]	recovery rate [%]
GelMA30	0.82	1.22	0.01	0.04	
GelMA60	0.41	4.62	0.73	0.07	
GelMA30GA	0.28	4.05	0.43	0.08	
GelMA60GA	0.92	0.05	0.01	0.02	
GelMA30GA-2.5Fe	0.42	76	0.80	0.74	
GelMA60GA-2.5Fe	0.26	4.16	0.16	0.04	
GelMA30GA-5Fe	0.03	7940	276	83	∼73
GelMA60GA-5Fe	0.23	6371	21	21	∼72

### Pre-Evaluation of Printability

**Figure 5:**
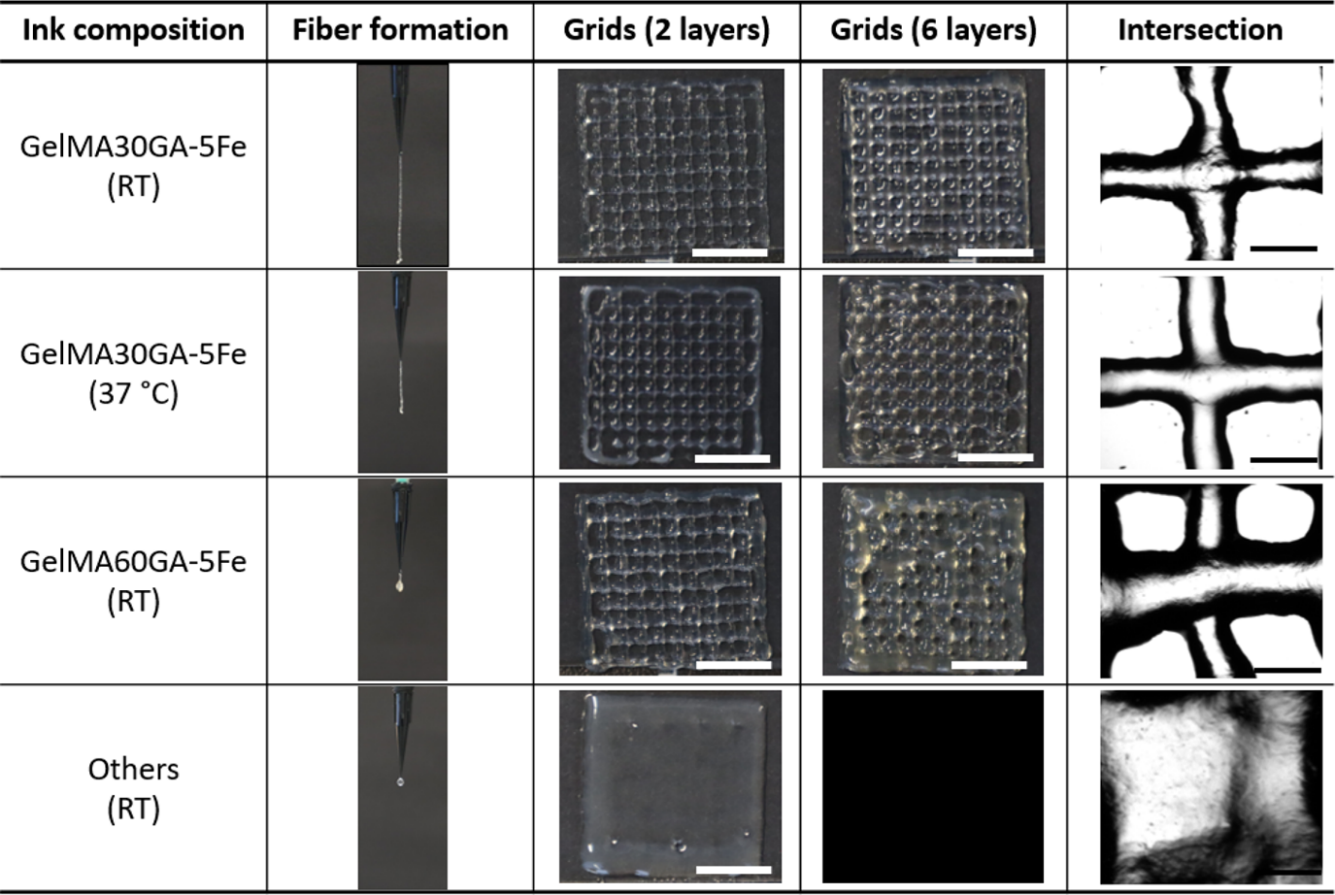
Prescreening of biomaterial inks: fiber formation, two-layered and six-layered printed grids, and close-up of filament intersections. Scale bar = 10 mm (white), 1 mm (black).

**Figure 6:**
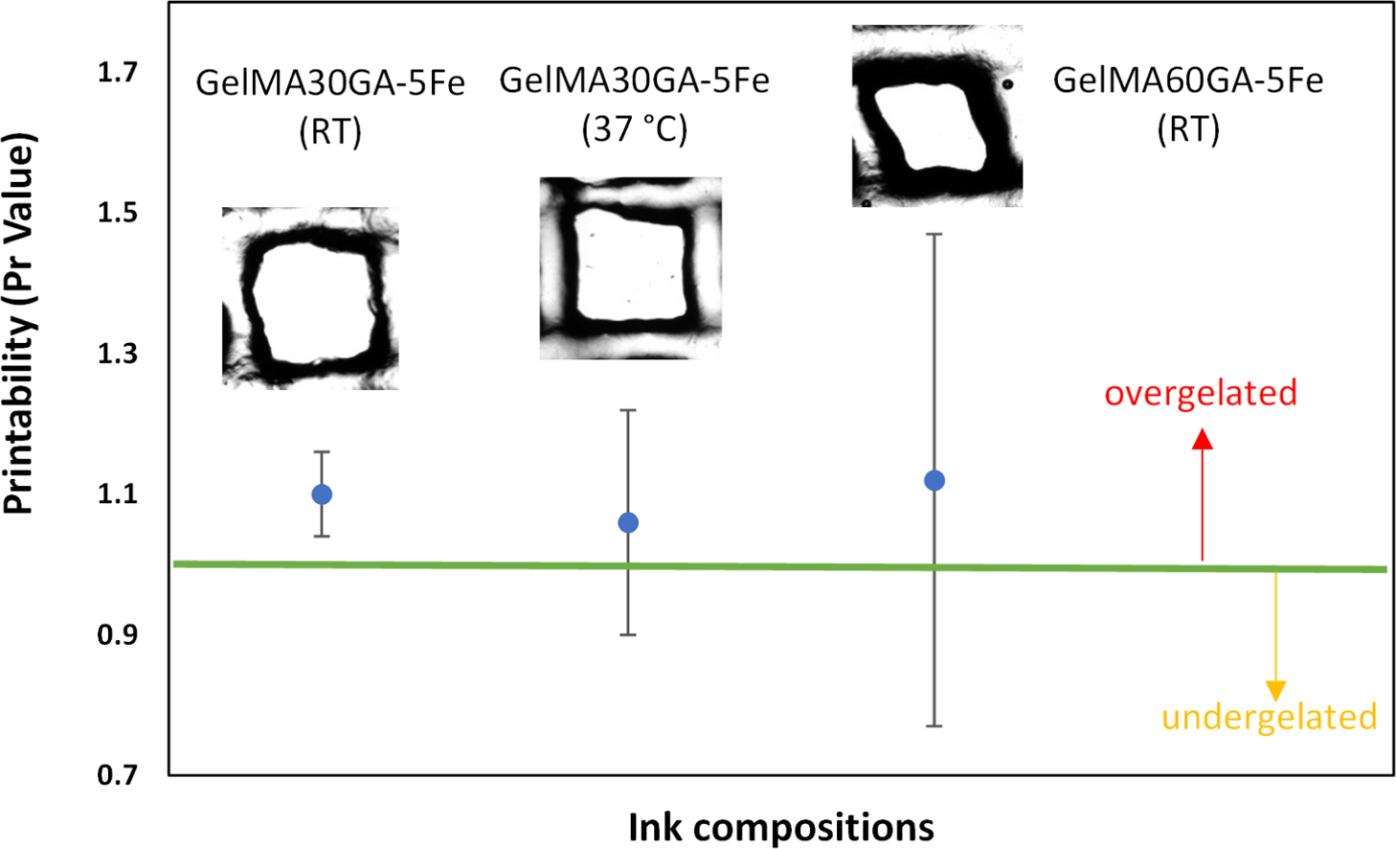
Calculated Pr values for the determination of the actual printability of GelMA30GA-5Fe at RT, GelMA30GA-5Fe at 37 °C, and GelMA60GA-5Fe at RT. The green line indicates the perfect printability value of 1. The Pr values are represented as mean ± standard deviation (*n* = 20).

As shown in Figure 5, the prescreened inks, GelMA30GA-5Fe and GelMA60GA-5Fe, were printed into grid structures at RT. In addition, GelMA30GA-5Fe was also printed at 37 °C. GelMA60GA-5Fe was extruded as small fragments formed from the cross-linked hydrogel, resulting in random-sized filaments when fabricating multiple stacked layers. GelMA30GA-5Fe was fabricated with high resolution when printed into two or six layers. At the elevated temperature, the geometry of the grids and filaments was not constant; instead, multilayered constructs started to collapse. The images of filament intersections showed that all pre-screened inks were able to stack without merging. The printability (Pr value) was calculated from the pore geometry inside the grids. Figure 6 shows that the average Pr values of all inks were close to each other (Pr = 1.1), had irregular shapes, and fell into the over-gelation area of the graph. However, the standard deviation values increased when the methacrylate modification was higher, supported by the filament formation data and the printing results. Also, the temperature-responsive behavior of GelMA resulted in irregularly shaped multilayered constructs (Figure 5, GelMA30GA-5Fe at 37 °C).

### 3D Printed Structures

**Figure 7:**
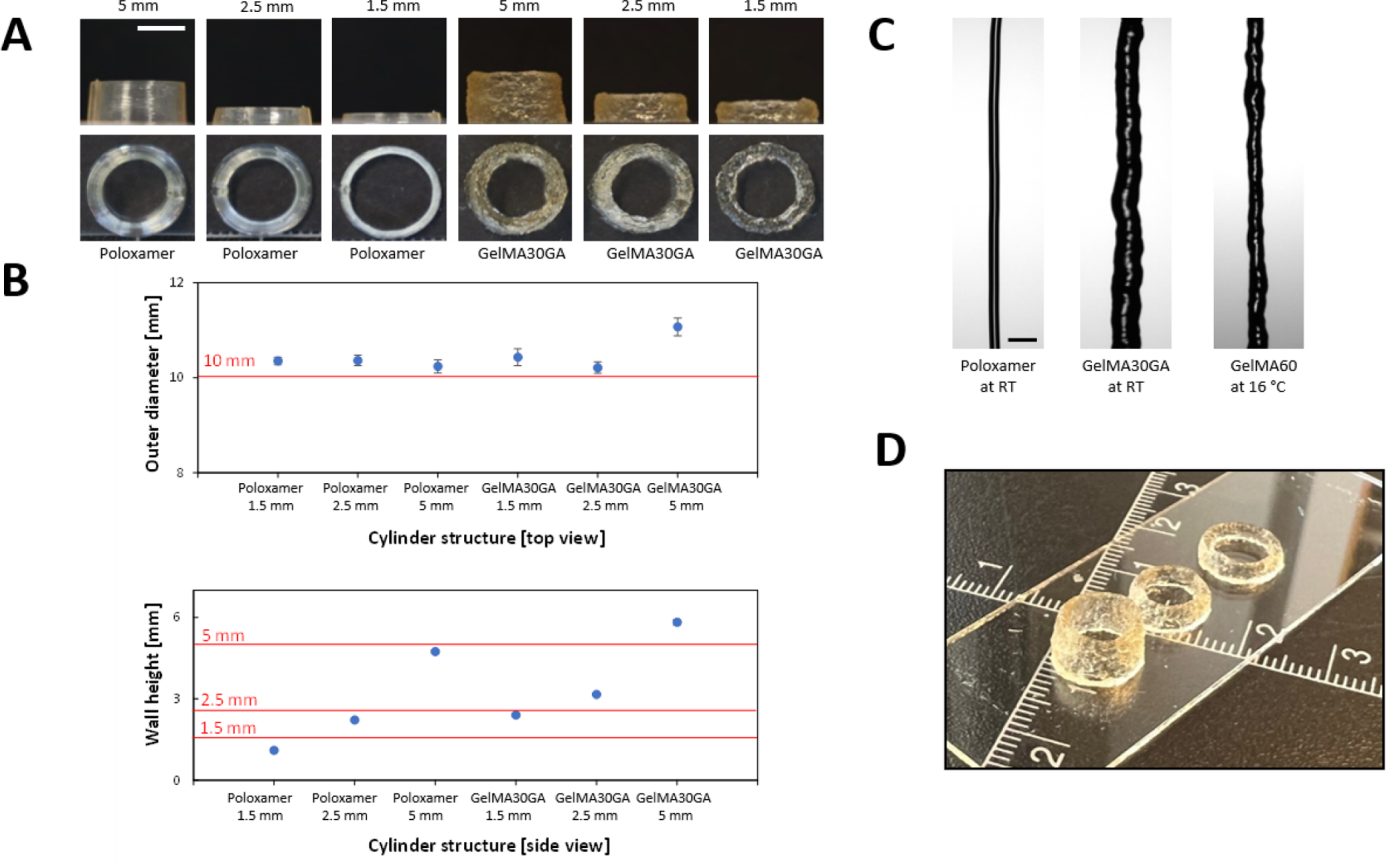
3D printed structures of GelMA30GA-5Fe ink and control material (poloxamer). (A) Top and side views of printed structures with 1.5, 2.5, and 5 mm wall heights (theoretical heights from CAD models). Scale bar = 5 mm (white). (B) Measured outer diameters and wall heights of cylinders compared to the heights from the CAD model (red lines). (C) Extruded filaments to observe filament widths of poloxamer at RT, GelMA30GA-5Fe at RT, and 5% w/v GelMA60 at 16 °C. Scale bar = 0.5 mm (black). (D) Examples of printed cylinders of GelMA30GA-5Fe. The ruler scale is in centimeter.

The CAD models of cylinders had a wall height of 1.5, 2.5, or 5 mm and consisted of 9, 16, or 33 layers. The dimensions of GelMA30-5Fe printed structures, including outer diameters and heights (Figure 7A,B), were measured and compared to printed Poloxamer to calculate the printing accuracy. All outer diameters of cylinders were consistent across all the structures (10.1–10.3 mm compared to 10 mm of the CAD model), except for the 5 mm GelMA30GA-5Fe cylinder, which has a measured height of 11 mm. In Figure 7C, the CA camera images show that the filament width of Poloxamer was close to the nozzle orifice, which was 0.2 mm. The filament width of GelMA30GA-5Fe swelled after being extruded (0.45 mm), resulting in higher cylinders. The shape fidelity of the 3D construct was confirmed by further characterization of filament shapes. The printed cylinders from three ink types, Poloxamer (RT), GelMA30GA-5Fe (RT), and GelMA60 (16 °C), were observed to confirm the printed structure resolution. The comparison of the top and side views of the structures showed that GelMA30GA-5Fe was able to maintain good shape fidelity and enabled the printing of multilayered 3D constructs (Figure 7A). Figure 7D illustrates the overview of all printed cylinders.

### Viscoelastic Properties

**Figure 8:**
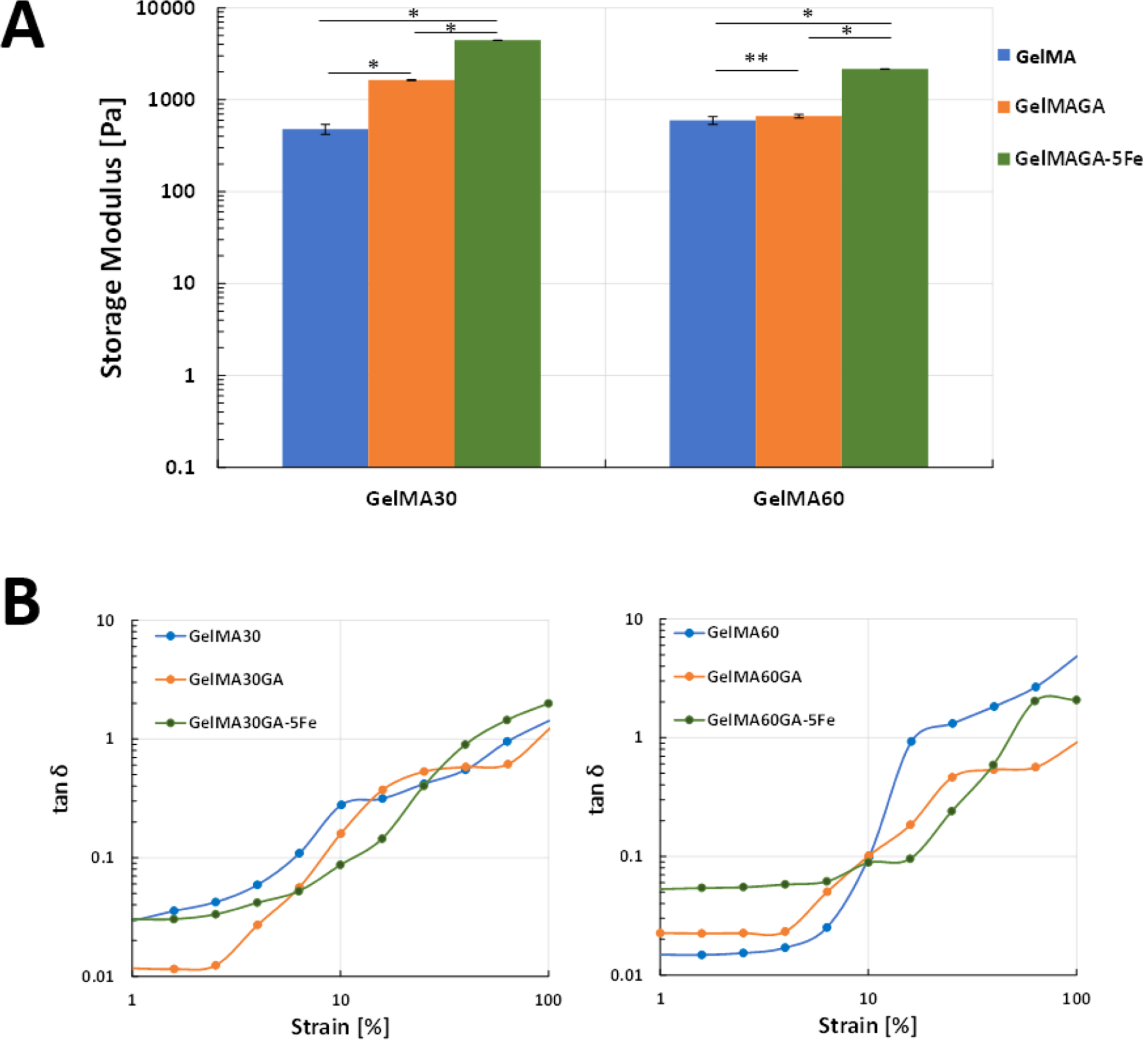
Oscillatory measurements of all hydrogel samples: GelMA30, GelMA60, GelMA30GA, GelMA60GA, GelMA30GA-5Fe, and GelMA60GA-5Fe, measured *via* frequency and amplitude sweeps at RT. The error bars indicate the standard deviation of storage modulus for each ink, presented as mean ± SD (*n* = 10, *p < 0.05, **insignificant). (A) Storage moduli of hydrogels in frequency sweep, (B) tan δ value, calculated from the ratio between *G*′ and *G*″ from amplitude sweep to observe the elasticity of hydrogels.

The oscillatory measurement data demonstrated that the addition of GA in GelMA30 led to a significant increase in the storage modulus values, but no such increase was observed for GelMA60 versus GelMA60GA (Figure 8A). The inks with FeCl_3_ yielded a significantly higher storage modulus compared to the samples without GA and FeCl_3_. Figure 8B shows that dual cross-linking using photocross-linking with FeCl_3_ resulted in higher elasticity than photocross-linking GelMA and GelMAGA without FeCl_3_. At low strain (1%), all samples displayed higher storage modulus and with increasing strain (100%), the storage modulus was reduced, while the loss modulus increased. The results were supported by the tan δ value, which is the ratio between *G*′ and *G*″ in Table 3. The tan δ value gave values significantly lower than 1. The tan δ value of GelMA30GA-5Fe and GelMA60GA-5Fe slowly increased after 10% strain compared to GelMA and GelMAGA (<5% strain), indicating that the gels were highly elastic. The average mesh sizes (ξ) and cross-linking densities (*n*_e_) were calculated using eqs 4 and 5 and are shown in Table 3. GA-functionalized GelMA hydrogels had higher cross-linking density, which led to stiffer hydrogels and smaller average mesh size. On the other hand, GelMA60 and GelMA60GA did not show a significant improvement in *G*′, resulting in an insignificant difference in the cross-linking densities and average mesh sizes (*p* > 0.05). In comparison to all other ink formulations, GelMAGA with FeCl_3_ had a significantly smaller average mesh size due to the higher values of *G*′ and cross-linking densities (*p* < 0.05).

**Table 3:** Storage and Loss Moduli, Calculated Average Mesh Sizes (ξ), and Cross-linking Densities (*n*_e_) for the Investigated Ink Compositions

	*G*′ [Pa]	*G″* [Pa]	ξ [nm]	*n*_*e*_[mol/m^3^]
GelMA30	478 ± 7	14 ± 2	20.52	0.19
GelMA60	594 ± 5	8 ± 1	19.06	0.24
GelMA30GA	1631 ± 26	34 ± 5	13.62	0.66
GelMA60GA	662 ± 30	15 ± 1	18.38	0.27
GelMA30GA-5Fe	4454 ± 38	135 ± 9	9.75	1.87
GelMA60GA-5Fe	2166 ± 43	115 ± 1	12.38	0.87

### Stability Test: Swelling Behavior and Dissolution Test

**Figure 9:**
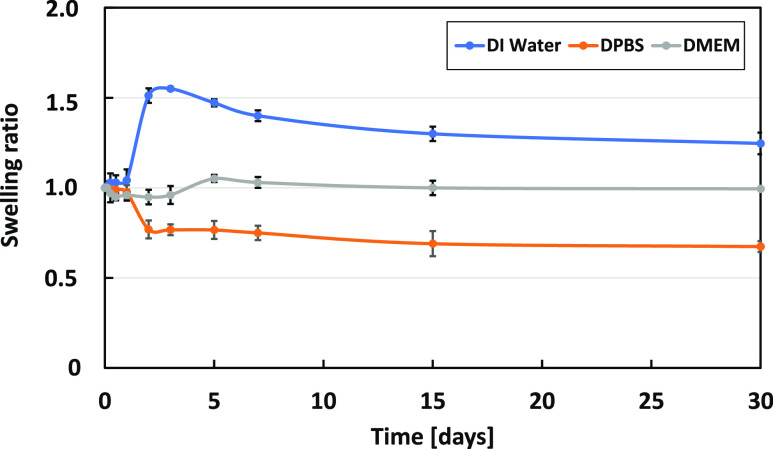
Stability test of 3D printed GelMA30GA-5Fe structures in DI water, DPBS, and DMEM for 30 days. The mean (*n* = 3) and standard deviation are shown.

The results of the stability test of the printed constructs, including swelling behavior in water and dissolution test in DPBS and DMEM, are presented in Figure 9. GelMAGA showed rapid initial swelling in water during the first 3 days (swelling ratio 1.51 ± 0.03), followed by slow degradation after the following days, but ultimately it remained stable for 1 month (swelling ratio 1.24 ± 0.22). In addition, the samples in DMEM absorbed a small amount of buffer and remained stable with swelling ratios of 1.05 ± 0.05 and 0.93 ± 0.03, respectively. However, the hydrogel in DPBS dissolved over a period of 7 days (swelling ratio 0.95 ± 0.05) and remained stable until the end of the observation.

### Tissue Adhesive Properties

A tack test was performed to investigate the tissue adhesive properties of different inks using the chicken skin and porcine muscle. Both GelMA and GelMAGA showed tissue adhesive properties (Figure S5). However, GA-modified GelMA required greater pull-up force from the *in situ* photocross-linked hydrogels (higher negative force) than GelMA.

### Antioxidant Properties

The DPPH reagent underwent a visual change in color from deep purple to deep orange in GelMA30GA and GelMA60GA because of the antioxidant properties imparted by GA. The UV–vis spectroscopy measurement of 2 mg/mL GelMA30GA and GelMA60GA in the presence of DPPH displayed 26 and 37% reduction (Figure S6) in absorption, indicating potential antioxidant properties.

## Discussion

The printability of biomaterial inks/bioinks is highly dependent on viscosity and flow behavior. The common approaches to improve the printability of GelMA are to increase the polymer concentration, lower the printing temperature, or mix it with other polymers.^8^ GelMA has been printed on its own with a concentration higher than 10% w/v at RT.^6,10,31^ However, high concentrations of polymers can result in reduced nutrient and oxygen transport for cells.^32^ Printing 5% w/v GelMA at low printing temperature (16–17 °C) could generate more cell injuries, and the temperature might not be homogeneously distributed throughout the cartridge, nozzle, and printing bed.^10,33^

To overcome the temperature-related issues, we synthesized GelMAGA from GelMA having two degrees of methacrylation (30 and 60%), followed by 10% GA conjugation to GelMA.^15,33^ Furthermore, the rheological properties were enhanced by pre-cross-linking with FeCl_3_*via* catechol–Fe^3+^ chelation, allowing lower polymer concentrations to be printable at RT or physiological temperature. Adding Fe^3+^ to GelMAGA inks can enhance the viscosity, providing primary cross-linking of the ink. After printing each layer, the ink was stabilized by photocross-linking (i.e., secondary cross-linking method). This sequential cross-linking approach significantly improves the printability of low-concentration GelMA-based bioinks (5% w/v).

All synthesized biomaterial inks were screened according to our pre-processing method. The prescreened results also showed that 0.5% FeCl_3_ in GelMAGA provided a favorable viscosity for the biomaterial inks, which were able to form a filament at RT due to noncovalent interactions of catechol–Fe^3+^ chelation. The coordination bonding between the trivalent ferric ions and hydroxyl groups of the GA leads to the formation of a loose hydrogel network and, hence, increases the viscosity of the inks.^34^ However, extruded GelMA60GA-5Fe filament was slightly overgelated, and it could not support its own weight in the air, resulting in a discontinuous filament. The concentration of 0.25% w/v FeCl_3_ in GelMAGA was not high enough to maintain the shape of the ink and led to droplet formation in the prescreening tests. Furthermore, the prescreening test showed that GelMA30GA-5Fe had good filament formation and stackability.

In general, printable biomaterial inks/bioinks are shear-thinning, having a viscosity that decreases with an increase in shear rate.^35−39^ The inks should exhibit yield stress, that is, have appropriate shear stress that must be overcome to make the ink flow. However, too high shear stress can cause the ink to burst and cause cell damage when printing with cells. Also, the initial viscosity value should be recovered at least up to 80% of the original level within seconds after printing.^36,40^ The temperature sweep of the flow mode showed that the viscosity of GelMAGA with Fe^3+^ was not much affected by the temperature change from RT to 37 °C. It indicates that primary cross-linking of Fe^3+^ can stabilize the ink at an elevated temperature. Instead, the viscosity of the inks slowly decreased after 4 °C compared to GelMA or GelMAGA. In general, increasing the gelatin modification degree decreases the physical interactions between the macromolecules, resulting in lower precursor viscosities and lower sol–gel transition temperatures.^41^ The results show that GelMA60GA ink displayed less thermostability compared to GelMA30GA. Similar behavior has been described previously: high modification of GelMA disturbs the triple helix structure due to reduced ionic and dipole–dipole interaction between gelatin molecules, resulting in a looser physical network that leads to the lower thermostability of the hydrogel network.^41^

The values of shear-thinning coefficients and yield stress were used to explain printability. GelMA30GA-5Fe and GelMA60GA-5Fe had high zero shear viscosity and did not flow immediately after the beginning of the measurement. Thus, both inks possessed yield stress, confirmed by the plotting of the Herschel–Bulkley model. All GelMAGA inks with and without Fe^3+^ were shear-thinning, supported by the Power-law model results, giving *n* < 1. However, our previously published study indicates that the *n* value should be lower than 0.2 to ensure high printability.^22^ In addition, low zero shear viscosity can result in poor fiber formation because of a lack of shape fidelity after being extruded from the nozzle.^42^Figure 4C shows that the viscosity of GelMA60GA-5Fe dropped sharply when the geometry started to move. This may be related to overgelation of the Fe^3+^ network. The recovery behavior tests demonstrated that GelMA30GA-5Fe and GelMA60GA-5Fe could recover 70% of their initial viscosity after removing the high shear. This results from the reversible interaction between GA and Fe^3+^ ions.^43^ We interpreted that the multiple long-range ionic interactions due to quadruple hydrogen bonds between Fe^3+^ and the phenolic groups resulted in favorable shear-thinning and recovery behavior of the inks.^44^

Based on the prescreening and rheological measurements, GelMA30GA-5Fe and GelMA60GA-5Fe were chosen to be evaluated for their printability (Pr) using a 3D bioprinter. Bioinks with excellent printability will exhibit constant shape and square pores in the printed grid structures. The calculated Pr values were similar, but the standard deviations varied, indicating the random pore geometries in GelMA60-5Fe grids. On the contrary, GelMA30-5Fe showed almost similar Pr values at RT and 37 °C. When printed into six-layered grids, GelMA30GA-5Fe at 37 °C and GelMA60GA-5Fe at RT resulted in irregular grid structures, which collapsed during printing.

GelMA30GA-5Fe was chosen to be printed into cylinders as well and further studied for its mechanical properties and stability. Previous studies have shown that UV light might not penetrate through the 3D structures, but photocross-linking in a layer-by-layer manner during the printing can increase the homogeneity of the printed structures.^45^ The measured cylinder diameters were quite similar to the control, but the wall heights differed from the CAD model, which probably resulted from the die swelling of the filament after being extruded from the nozzle. The inaccuracy of the printed 3D constructs was also supported by the filament shape characterization showing die swelling of GelMA30GA-5Fe (RT). By comparing the top and side views of the cylinders, it is obvious that GelMA30GA-5Fe was still able to maintain good shape fidelity and enabled multilayered printing.

According to the oscillatory measurement, the storage moduli of GelMA30G-5Fe and GelMA60GA-5Fe were significantly higher than that for the ink without GA and Fe^3+^. In addition, GA and Fe^3+^ improved the elasticity of the resulting GelMA hydrogels, as shown in Figure 8B, because of the double network formed between GA and Fe^3+^. The interconnectivity and integrity due to photocross-linking and catechol–Fe^3+^ chelation provided a more stable network than in a single network GelMA (single photocross-linking).^21,26,46^ Dual cross-linking in the GelMAGA-Fe hydrogels yielded a smaller mesh size and higher cross-linking density as compared to the single network in the GelMA hydrogel. However, the denser polymer network can limit the transport of oxygen and nutrients to the cells.^30,47^

Swelling and dissolution tests were performed to evaluate the stability of GelMAGA printed structures in water, DPBS, and DMEM under a physiological environment.^47,48^ The structures were stable for over a month in the aqueous solution at 37 °C, with a slight change during the first 2 days. In the previously reported studies, the weight of GelMA hydrogels increased by almost 60% in PBS after just 24 h.^6,10,48^ In comparison, our GelMA30GA-5Fe swelled less than 10% and the printed structures were able to maintain internal and external architecture until the end of the observation period. As assumed, the cross-linking density and average mesh size influenced the swelling capacity of the hydrogel.^30^ The higher cross-linking density resulted from the dual network formation leading to a reduction in water absorption.^49^ In addition, we observed that GelMAGA-Fe displays considerable tissue adhesive and antioxidant properties, as shown in Figures S4 and S5. Adhesive biomaterial inks can be useful as a printable glue, and they expand the bioink application possibilities, enabling, for example, printing directly to the defect site for wound dressing purposes.^20,26^

## Conclusions

We developed a GA-functionalized GelMA-based biomaterial ink utilizing a two-step sequential cross-linking approach: metal–ligand complexation followed by photocross-linking. The pre-cross-linked GA-modified GelMA with Fe^3+^ (GelMA30GA-5Fe) showed higher viscosity and better rheological profile than GelMA ink alone, resulting in superb printability. It was also printable into 3D constructs with good shape fidelity compared to the ink without a pre-cross-linker. The dual network achieved by catechol–Fe^3+^ chelation and photocross-linking also improved the elastic modulus in the hydrogels, compared to GelMA and GelMAGA. The printed structures of GelMA30GA-5Fe ink showed good stability and a low swelling ratio in the physiological environment over a month. In addition, GA provided tissue adhesion and antioxidant properties. The catechol-based adhesive printable inks can offer the tissue-engineered scaffolds better attachment on the surface of target organs or tissues without using additional glue. Moreover, the GA-modified GelMA ink opens up new possibilities for wound dressing materials that can be utilized for *in situ* bioprinting at the defect site.

## Supporting Information Available

Calibration curve for residual amine concentration using glycine standard; measured UV light intensity versus distance from the light source; additional UV spectra of Gelatin type A, GelMA, and GelMAGA; calculation of the degree of methacrylation and gallic acid modification; further confirmation of GelMAGA conjugation; tack test of GelMA30, GelMA60, GelMA30GA, and GelMA60GA; and DPPH radical scavenging assay of GelMA30GA and GelMA60GA (PDF)

## Abbreviations

GelMA: methacrylated gelatin
MA: methacrylate
GA: gallic acid
UV: ultraviolet
RT: room temperature
*G*′: storage modulus
*G*″: loss modulus
PI: photoinitiator
CAD: computer-aided design
DI: deionized
