# Supplementary material for: Sequential Cross-linking of Gallic Acid-Functionalized GelMA-Based Bioinks with Enhanced Printability for Extrusion-Based 3D Bioprinting
Source: Biomacromolecules. 2022 Dec 22;24(1):502–14. doi: 10.1021/acs.biomac.2c01418 (PMC9832479; doi:10.1021/acs.biomac.2c01418)
Supplement: Supplementary file 1 — bm2c01418_si_001.pdf [file bm2c01418_si_001.pdf]

Supporting Information

Sequential Crosslinking of Gallic Acid Functionalized GelMA-based Bioinks with Enhanced Printability for Extrusion-based 3D Bioprinting

Hatai Jongprasitkul[a], Sanna Turunen [a,b], Vijay Singh Parihar[a,], Minna Kellomäki[a]*

[a]Biomaterials and Tissue Engineering Group, BioMediTech, Faculty of Medicine and Health Technology, Tampere University, 33720 Tampere, Finland

[b]Brinter Ltd, 20520 Turku, Finland

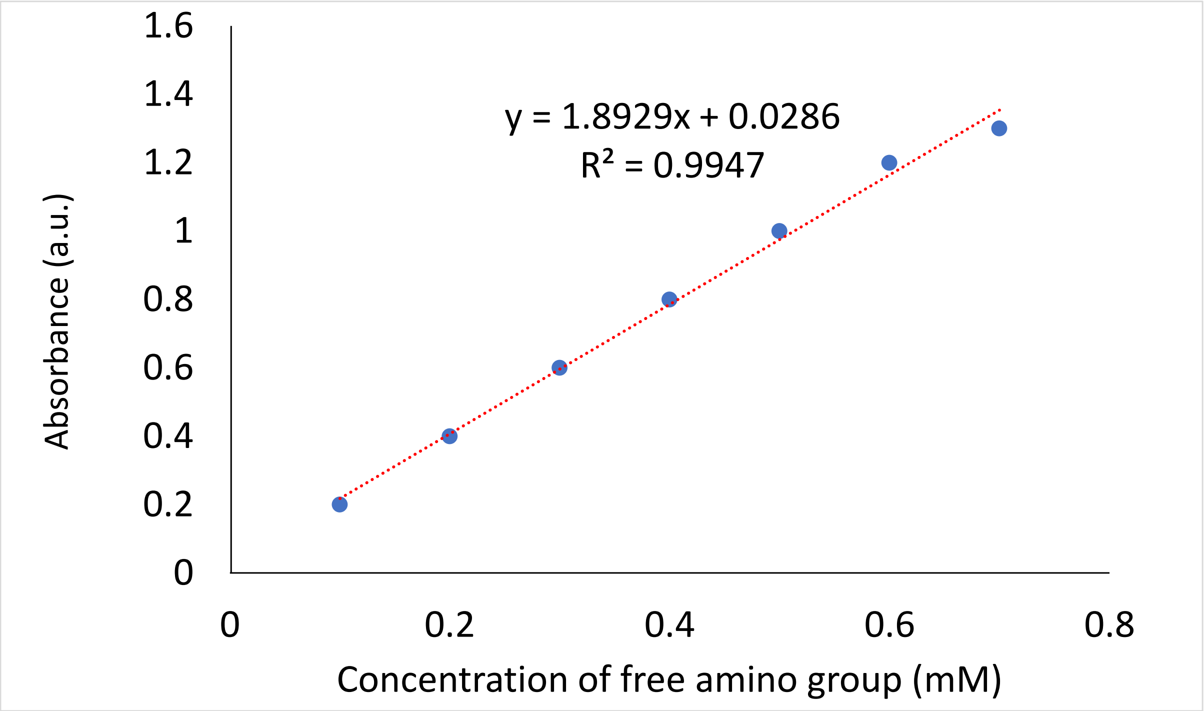

Figure S1: Calibration curve for residual amine concentration using glycine standard by varying concentrations and under identical conditions to Gelatin A, GelMA and GelMAGA.

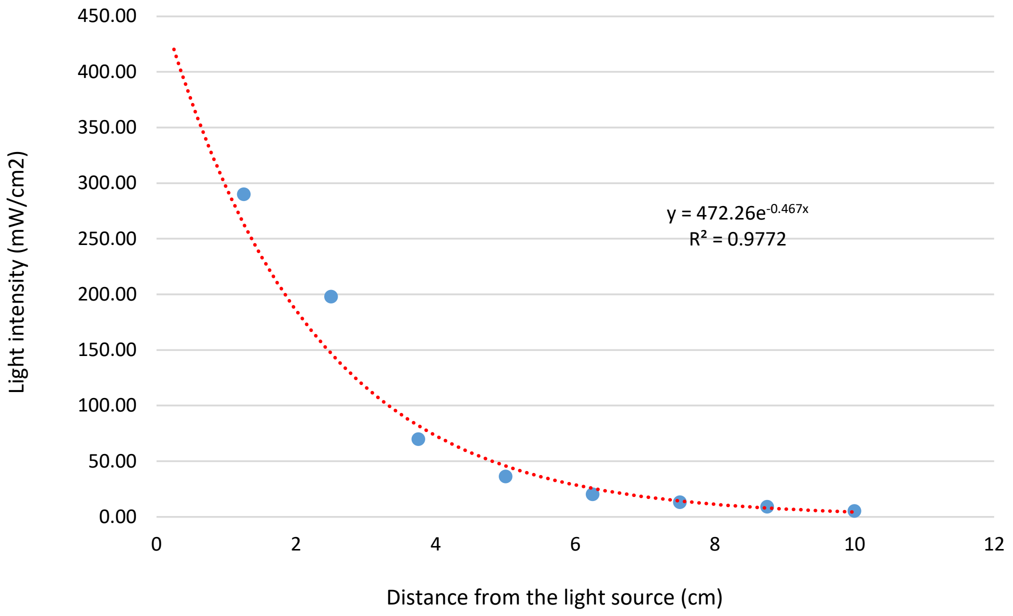

Figure S2: The measured UV light intensity vs distance from the light source.

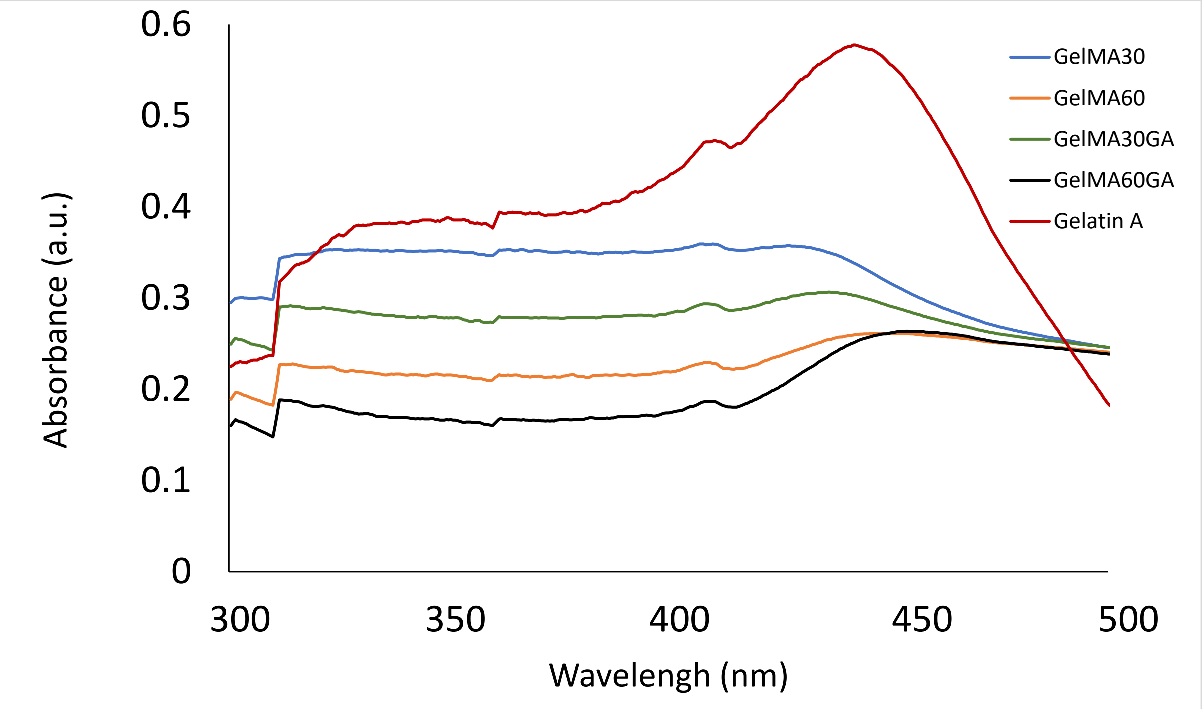

Figure S3: UV-Vis spectra of Gelatin type A, GelMA and GelMAGA. Gelatin Type A contained 0.29 mmol/g of free amines according to the calibration curve (Figure S1).

Table S1: The degree of methacrylation and gallic acid modification based on free amine concentration.

Materials	Abs.	Free amines		Methacrylate		Gallic acid	
		mmol/g	%	mmol/g	%	mmol/g	%
Gelatin type A	0.46	0.29	100	0	0	0	0
GelMA30	0.33	0.20	68.96	0.09	31	0	0
GelMA60	0.21	0.11	37.93	0.18	64	0	0
GelMA30GA	0.29	0.17	58.62			0.031	10.34
GelMA60GA	0.15	0.08	27.96			0.028	9.97

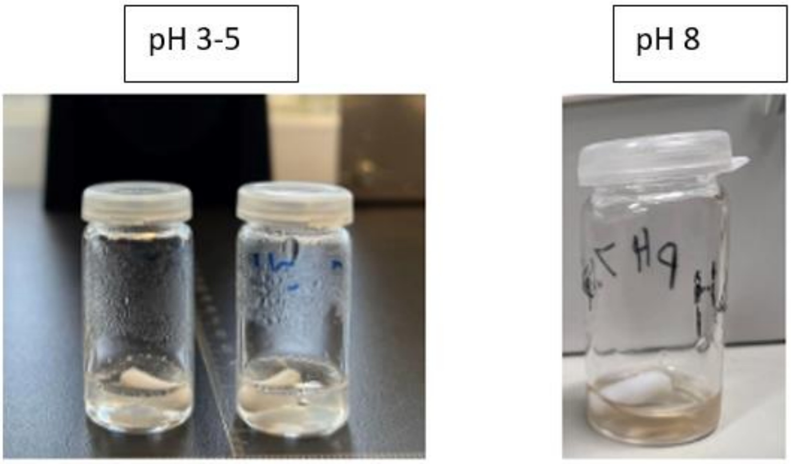

Figure S4: The further confirmation of GelMAGA conjugation, the solution turned light brown at pH 8.

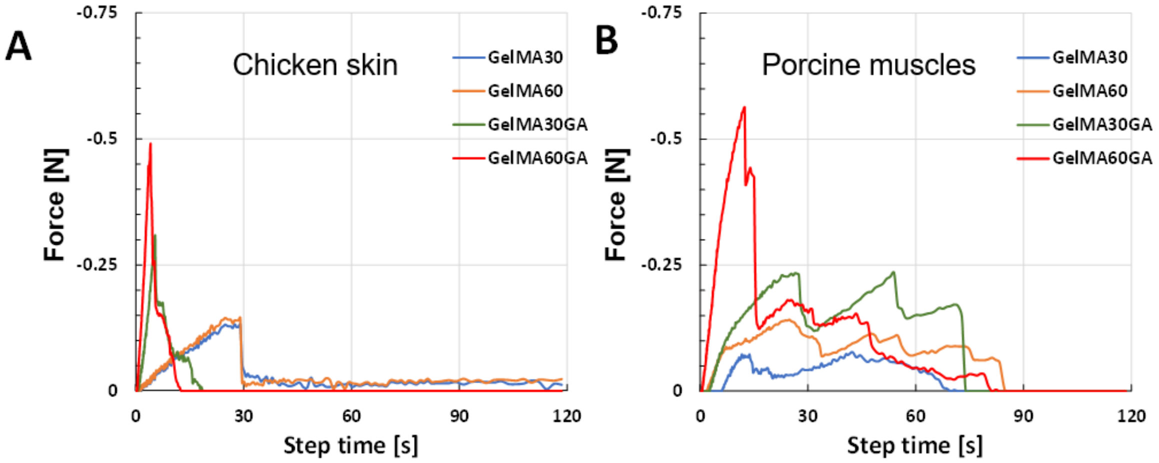

Figure S5: Measured tissue adhesive force for GelMA30, GelMA60, GelMA30GA and GelMA60GA with (A) chicken skin and (B) porcine muscles. The mean (n=3) of the measurements is shown. Standard deviations are not presented as they were smaller than the curve because the data was measured in milliseconds.

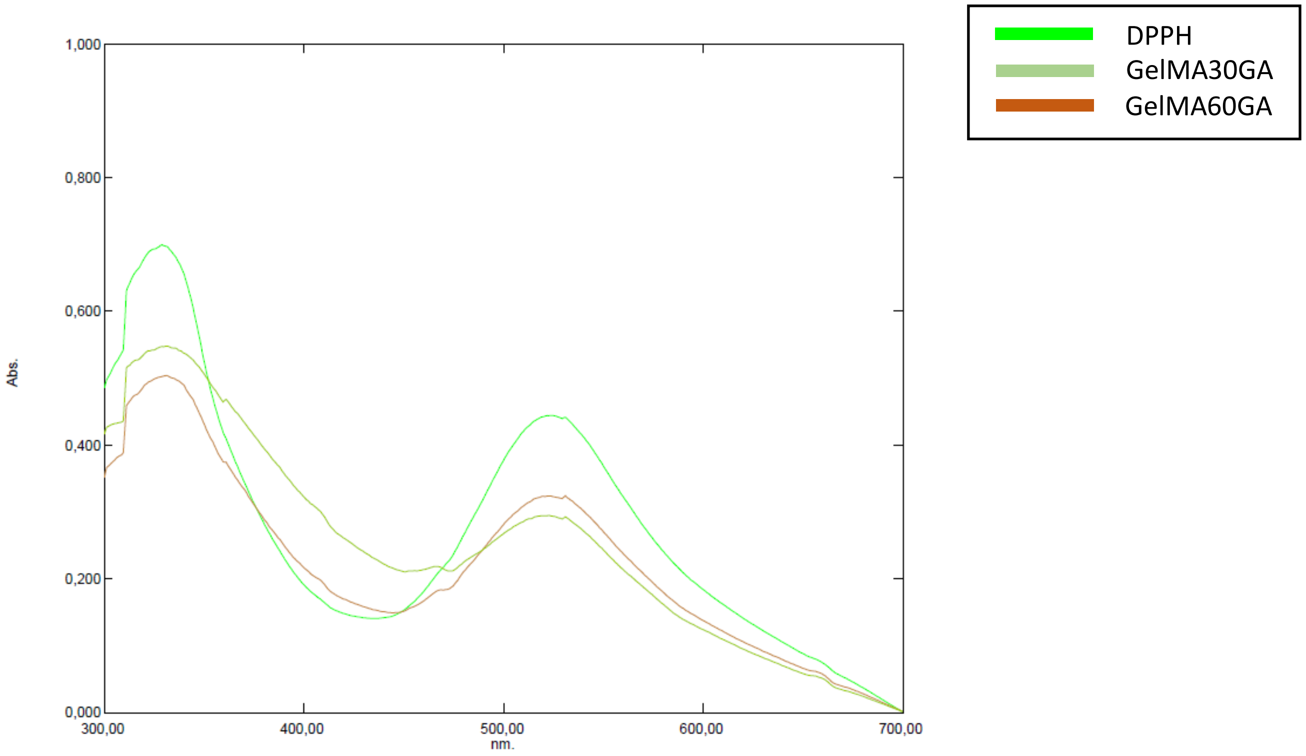

Figure S6: The antioxidant property of GelMA30GA (yellowish green) and GelMA60GA (brown), determined via DPPH (green) radical scavenging assay.
